# Numerical Analysis of Particulate Migration Behavior within Molten Pool during TIG-Assisted Droplet Deposition Manufacturing of SiC Particle-Reinforced Aluminum Matrix Composites

**DOI:** 10.3390/ma14092430

**Published:** 2021-05-07

**Authors:** Yubin Zhang, Jun Du, Huafeng Wang, Hua Li, Xiaoyun Zhao

**Affiliations:** College of Digital Technology and Engineering, Ningbo University of Finance & Economics, Ningbo 315175, China; zhangyubin@nbufe.edu.cn (Y.Z.); wanghuafeng@nbufe.edu.cn (H.W.); lihua@nbufe.edu.cn (H.L.); zhaoxiaoyun@nbufe.edu.cn (X.Z.)

**Keywords:** particle migration behaviors, droplet deposition manufacturing, SiC particle-reinforced aluminum matrix composites, molten pool

## Abstract

A transient three-dimensional (3D) numerical model was established to illustrate the heat transfer, fluid flow and particle migration behaviors in the molten pool during TIG-assisted droplet deposition manufacturing (DDM) of SiC particle-reinforced aluminum matrix composites (AMCs). The effect of temperature-dependent physical properties and the interaction between the SiC reinforcement and the liquid metal matrix were considered. A double-ellipsoidal volumetric heat source model was adopted to simulate the energy interactions between the pulse square-wave variable polarity TIG welding arc and the moving substrate. Free surface fluctuations of molten pool due to arc force and sequential droplet impact are calculated with volume of fluid (VOF) method in a fixed Eulerian structured mesh. The numerical model, capable of capturing the impact, simultaneous spread, and phase change of the droplets as well as the motion trajectory and terminate distribution state of the reinforcement particles, is key tool to understand the formation mechanism of the TIG-assisted DDM of SiC particle-reinforced AMCs. The numerical model was validated by the metallographic observations, and the calculated particle distribution and solidification morphology of deposited layer agree well with the experimental measurements.

## 1. Introduction

Compared to unreinforced metal matrix, ceramic-reinforced metal matrix composites (MMCs) can be utilized in extreme working conditions and some high-end engineering industries (i.e., automotive, aerospace, etc.) due to their higher mechanical, thermal, and fatigue properties at elevated temperatures [1]. The research and application of these materials such as particle-reinforced aluminum matrix composites (AMCs) will not only significantly expand the scope of potential applications but also achieve their economic potential [2]. Particle-reinforced AMCs are generally prepared by powder metallurgical technique [3] and melt-metallurgical production method [4,5,6]. Reinforced AMCs have the improved mechanical properties, such as the tensile strength [7], the yield strength [8], and the wear resistance [9]. However, it is difficult for the abovementioned preparation methods of particle-reinforced AMCs to fabricate complex-shaped components [10], but also, they have problems of high cost and energy consumptions.

Additive manufacturing (AM) is the manufacturing process of components through layer-based material consolidation (ASTM, 2012). Recently, the emerging manufacturing technology with tailorable composition and properties, lowered cost and energy usage, and high design flexibility has gained popularity in the fabrication of high-performance materials. Different research groups investigated the feasibility to use direct metal laser sintering (DMLS) to achieve Al-based metal matrix composites [11,12]. SiC particles with different size and volume fraction were employed to analyze the behaviors of the composite, demonstrating that cracking occurs during the DMLS of these composites. It is found that obvious reaction occurs between aluminum melt and SiC particles, leading to the formation of Al_4_SiC_4_ [13]. It is also proved that in any case, the presence of the ceramic reinforcement on hardness is evident. Meanwhile, there is a growing research to develop MMCs via selective laser melting (SLM) process [14,15]. However, due to the low absorption of the laser and the high thermal conductivity of the aluminum alloys, the deposition rate of aluminum alloys and Al-based composites via laser AM is very low [16]. Although wire arc additive manufacturing (WAAM) has also proven its capability to produce medium-to-large-scale aluminum components, aluminum-based filler wire containing ceramic particles are rarely seen. Therefore, it is necessary to develop a novel and highly efficient forming method to realize the additive manufacturing of complex AMCs components.

In this study, a novel hybrid additive manufacturing process, termed as TIG-assisted droplet deposition manufacturing, is proposed to fabricate SiC particle-reinforced AMCs components, the process principle of the additive manufacturing technique will be described in detail in Section 2. A 3D numerical simulation model based on finite volume method (FVM) was proposed to understand and analyze the molten pool dynamics and particle-melt interactions within the molten pool during the TIG-assisted DDM of SiC particle-reinforced AMCs. The resultant distribution state of SiC particles in the solidified aluminum matrix was experimentally examined, which is in a good agreement with the results calculated by simulation.

## 2. Principle of TIG-Assisted DDM Process

**Figure 1:**
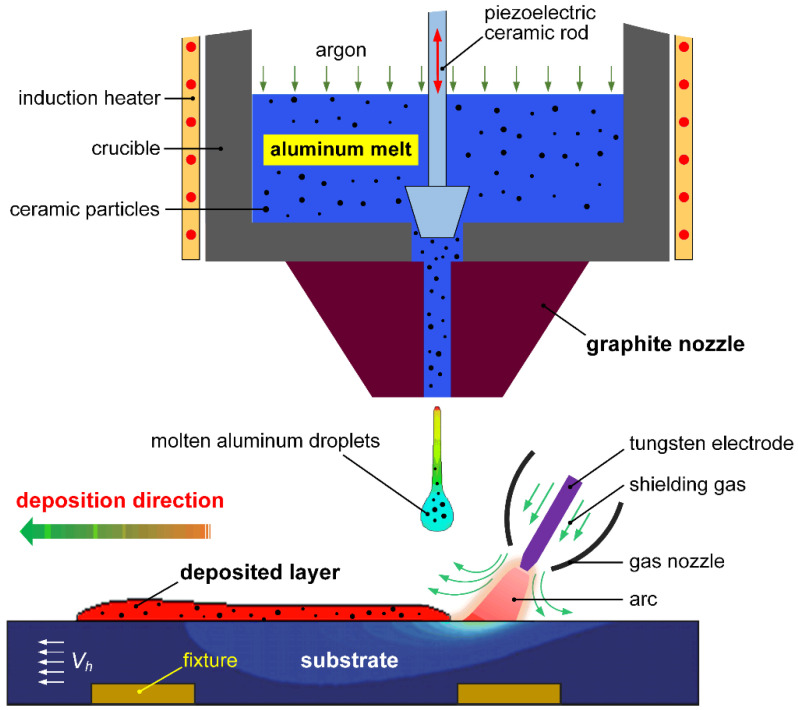
Schematic diagram of the TIG-assisted DDM process of particulate reinforced AMCs.

Figure 1 shows the schematic illustration of the TIG-assisted droplet deposition manufacturing process. The equipment of TIG-assisted DDM includes an induction-heated crucible, a piezoelectric ceramic rod, a gas protection device, and a 3D mobile platform. The TIG-assisted process can produce ceramic-reinforced AMCs components by synchronized controlling the 3D mobile platform and the injected molten aluminum droplets with ceramic reinforcements. A programmable multi-axis controller (PMAC) is used to control the motion of the mobile platform. A high-dynamic welding camera was applied to monitor the forming process. Under the combined action of the high frequency vibration by the piezoelectric ceramic rod, surface tension, and hydrostatic pressure, the molten aluminum will flow through the inner flow channel of graphite nozzle and to be injected in form of successive droplets. When the injected molten aluminum droplets impact and contact with the molten pool formed on the substrate by the pulsed variable polarity TIG welding arc, coalescence, spontaneous spreading and non-equilibrium solidification would take place. Thus, a metallurgical bonding can be achieved. The solidified deposition layers of reinforced AMCs always keep synchronous with the substrate. Variable polarity TIG welding arc has a two-fold effect in the TIG-assisted DDM: First, the oxides on the surface of aluminum alloy substrate could be removed timely. Fortunately, the VP-TIG welding arc can clean oxide film timely. Second, sound metallurgical bonding between the deposited layers could be achieved by applying the preceding VP-TIG welding arc, which substantially improve the high-temperature wettability and spreadability of molten aluminum droplets on the surface of previous deposited layers or substrate.

TIG-assisted DDM process has high deposition efficiency, low cost, great freedom in material selection, etc. It has a wide application prospects in aerospace, shipping, rail transportation and other fields. Compared to a single TIG welding and droplet deposition process, TIG-assisted DDM involves more process parameters, and thus, relying on experience or trial-and-error methods to determine optimized process parameters will be a long cycle and high-cost problem. With the development of computer technology and numerical calculation methods, using computer simulation technology to study the TIG-assisted DDM process of particle-reinforced AMCs has become possible, which is helpful to reveal the forming mechanism and optimize process parameters. 

In this work, the transient thermal fluid fields and particles migration behavior during the deposition process were calculated and analyzed using a 3D finite volume method model. The molten pool dynamics and the kinetic characteristics of SiC particles within the molten pool were well understood by visualizing the thermo-fluid field. This will provide theoretical basis for a deeper understanding of the mechanism and engineering application of TIG-assisted DDM.

## 3. Description of the Mathematical Model

### 3.1. Physical Description of TIG-Assisted DDM Process

TIG-assisted DDM of the particle-reinforced AMCs involves complex non-equilibrium metallurgical processes, which exhibits impacting and spreading of molten aluminum droplets with SiC particles, free-surface fluctuation, and unsteady heat transfer behaviors accompanied with solidification phase change. Arc force, buoyant force, and surface tension gradient force produce vortex flows in the melt, which directly affect the flow pattern and the associated physical metallurgy process. After cooling and solidification of the melt, the deposited layer has a definite distribution of SiC particles. The calculations of the morphology of deposited layer and the resultant distribution state of reinforcing particles are the important goals of this work.

### 3.2. Basic Assumptions

The melt motion induced by the impacting of molten aluminum droplets on the molten pool by the preceding TIG welding arc can be assumed to be laminar with a viscous incompressible heat-conducting fluid. In fluid dynamics, the Boussinesq approximation in a 3D Cartesian coordinate system is used in the field of buoyancy-driven flow. The influence of shielding gas on the flow pattern in the molten pool is ignored. The volume thermal expansion of the SiC particles is neglected. The fraction of particles in the liquid metal matrix is small, and thus, the particles collision with each other, and the influence of particles on the melt flow and surface tension can also be ignored. The thermal physical properties of the SiC particles are assumed to be constant.

### 3.3. Governing Equations

The governing equations describing the heat-mass transport of molten pool- impacting droplets with SiC particles include the mass, momentum, and energy conservation equations, as shown in Equations (1), (3), and (5). The morphology of SiC particle-reinforced AMCs deposited layers was calculated by adopting the volume of fluid (VOF) method, and the corresponding equation is shown in Equation (6).

Mass conservation equation,

(1) $$\nabla \cdot \vec{V}=\frac{{\dot{m}}_{s}}{\rho }$$

where $\vec{V}$ is the velocity vector of melt, ${\dot{m}}_{s}$ is the rate of the mass source of droplets, and *ρ* is density.

In the TIG-assisted DDM, molten aluminum droplets will be periodically added to the calculation model as a mass source term. It can be expressed as Equation (2).

(2) $$m_{s}=\frac{\rho_{d}}{\Delta t}$$

where *ρ_d_* is the droplet density, and ∆*t* is the time interval between adjacent droplets.

Momentum conservation equation,

(3) $$\frac{\partial \vec{V}}{\partial t}+\vec{V}\cdot \nabla \vec{V}=-\frac{1}{\rho }\nabla P+\mu_{k}\nabla^{2}\vec{V}-K\vec{V}+\frac{{\dot{m}}_{s}}{\rho }({\vec{V}}_{s}-\vec{V})+G$$

where *P* is the hydrodynamic pressure, *μ_k_* is the dynamic viscosity, ${\vec{V}}_{s}$ is the velocity vectors for the mass sources, *G* is the body acceleration due to body force (e.g., gravity, Lorentz force, and buoyancy forces), *K* is the deceleration coefficient in the semisolid zone, and *K* can be written as

(4) $$K=C\left[\frac{F_{s}^{2}}{{\left(1-F_{s}\right)}^{3}}+B^{*}\right]$$

where *C* is a constant representing semisolid zone morphology, *F_s_* is the solid fraction, B^*^ is the positive zero used to avoid division by zero. The Carman–Kozeny equation is used to model the flow in the semisolid zone, which is based on the derivation from the Darcy model and the assumption that this region is able to be treated as a porous media.

Energy conservation equation,

(5) $$\frac{\partial h}{\partial t}+\vec{V}\cdot \nabla h=\frac{1}{\rho }\nabla \cdot (k\nabla T)+{\dot{h}}_{s}+S_{q}$$

where *k* is the thermal conductivity of the material, *h* is the specific enthalpy, *T* is the temperature, *S_q_* is the other energy source terms, and ${\dot{h}}_{s}$ is the specific enthalpy source, which is associated with the mass source in Equation (1).

Volume of fluid (VOF) equation,

(6) $$\frac{\partial F}{\partial t}+\nabla \cdot (\vec{V}F)=F_{s}$$

where *F* is the volume fraction and *F_s_* is the volume source from the molten droplets.

Solidification model.

The enthalpy method is used to solve the melting and solidification problem. The internal energy equation of the system is

(7) $$\frac{\partial h}{\partial t}+v\cdot \nabla h=\frac{1}{\rho }\cdot (k\nabla T)$$

where *k* is the thermal conductivity of the material, *ρ* is density, *T* is time, and *h* is enthalpy; it can be expressed as

(8) $$h=\{\begin{array}{ll} \rho_{s}C_{s}T & \left(T\leq T_{s}\right)\\ h(T_{s})+h_{sl}\frac{T-T_{s}}{T_{l}-T_{s}} & \left(T_{s}\leq T\leq T_{l}\right)\\ h(T_{l})+\rho_{l}C_{l}(T-T_{l}) & \left(T_{l}\leq T\right) \end{array}$$

where *ρ_s_* and *ρ_l_* are solid and liquid densities; *C_s_* and *C_l_* are solid and liquid specific heats, respectively; *T_s_* and *T_l_* are solid and liquid temperatures of materials, and *h_sl_* is latent heat of fusion.

### 3.4. Driving Forces

The main driving forces acting on the molten pool are Lorentz force, arc pressure, surface tension, gravity, and buoyancy force. In this study, the arc pressure is approximated as Gaussian density distribution, as given below

(9) $$P_{arc}(x,y)=\frac{\mu_{0}I^{2}}{4\pi^{2}\sigma_{r}^{2}}\exp\left(-(\frac{r^{2}}{2\sigma_{r}^{2}})\right)$$

where *μ*_0_ is the magnetic permeability, and *σ_r_* is the distribution parameter of arc pressure.

The arc drag force acting on the molten pool surface can be represented as follows.

(10) $$P_{Drag}(r)=P_{Max}\sqrt{\frac{r}{r_{Shear}}}\exp-{\left(\frac{r}{r_{Shear}}\right)}^{2}$$

where *r_Shear_* is the distribution parameter of arc drag force.

The Lorentz force is considered by adopting the elliptically symmetric welding current density in the EMF model [17]. The equations relating EMF are listed as below.

(11) $$F_{x}=-J_{z}\times B_{\theta }\frac{x}{r_{a}}$$

(12) $$F_{z}=-J_{r}\times B_{\theta }$$

(13) $$J_{z}=\frac{I}{2\pi }\int_{0}^{\infty }\lambda J_{0}(\lambda r_{a})\exp(-\frac{\lambda^{2}\sigma_{x}}{2})\frac{\sinh\left[\lambda (c-z)\right]}{\sinh(\lambda c)}d\lambda$$

(14) $$J_{r}=\frac{I}{2\pi }\int_{0}^{\infty }\lambda J_{1}(\lambda r_{a})\exp(-\frac{\lambda^{2}\sigma_{x}}{2})\frac{\cosh\left[\lambda (c-z)\right]}{\sinh(\lambda c)}d\lambda$$

(15) $$B_{\theta }=\frac{\mu_{m}I}{2\pi }\int_{0}^{\infty }J_{1}(\lambda r_{a})\exp(-\frac{\lambda^{2}\sigma_{x}}{2})\frac{\sinh\left[\lambda (c-z)\right]}{\sinh(\lambda c)}d\lambda$$

(16) $$r_{a}=\sqrt{{\left(x-x_{0}\right)}^{2}+{\left[\frac{\sigma_{x}}{\sigma_{y}}(y-y_{0})\right]}^{2}}$$

where *I* is the welding current, *F_i_* are the components of Lorentz force in *i*-direction (*i* = *x*, *y*, *z*), *J_z_* and *J_r_* are the axial and radial current density, *B_θ_* is the angular component of magnetic field, *J*_0_ and *J*_1_ are the zero order and one order Bessel function, respectively.

The surface tension of the liquid AMCs can be expressed as a linear function of temperature:

(17) $$\gamma =\gamma_{0}+k_{\mathrm{sur}}(T-T_{0})$$

where *γ*_0_ is the surface tension coefficient at *T*_0_, *k*_sur_ is the temperature coefficient of surface tension.

### 3.5. Droplet Model with SiC Particles

In the TIG-assisted DDM process, the reinforcing phase in the deposited layer is introduced as the form of SiC particle within molten aluminum droplets. Droplet transition is achieved through the coupling drive of piezoelectric ceramic excitation and the flow channel of graphite nozzle. The relevant parameters of the droplets and the SiC particles are shown in Table 1. 

The motion control equation of the particle-reinforced phase is:

(18) $$\frac{du_{p}}{dt}=-\frac{1}{\rho_{p}}\nabla P+g+\beta (u-u^{\prime })|u-u^{\prime }|\frac{\rho }{\rho_{p}}$$

where $u^{\prime }=u_{p}+u_{diffusion}$, *u_p_* is the average velocity of SiC particles, *ρ_p_* is the average density of SiC particles, *g* is the total volume force to which SiC particles are subjected, *u* and *P* are respectively the flow velocity and pressure of the melt around the particle phase.

The motion trajectories and thermodynamics properties of SiC particles are described by Lagrangian method. It has been proved that the trajectory approach can be effectively used to simulate the transport properties of dispersed phase.

For an individual particle *p* with a radius *r_p_*, the velocity vector ${\vec{V}}_{p}=(u_{p},v_{p},w_{p})$ of the mass center and the volume-averaged temperature of the particle are considered. The related equations of motion and heat transfer for the individual spherical particle *p* are written in the form

(19) $$\frac{d{\vec{V}}_{p}}{dt}=\frac{3}{8}\frac{\rho C_{d}}{\rho_{p}r_{p}}\left|\vec{V}-{\vec{V}}_{p}\right|(\vec{V}-{\vec{V}}_{p})+\vec{g}\frac{\rho_{p}-\rho }{\rho_{p}}$$

(20) $$\frac{d{\vec{X}}_{p}}{dt}={\vec{V}}_{p}$$

(21) $$\rho_{p}\frac{4}{3}\pi r_{p}^{3}\frac{dH_{p}}{dt}=4\pi r_{p}^{2}\left[\frac{k_{m}}{2r_{p}}Nu_{p}(T_{p}-T)\right]$$

(22) $$T_{p}=\{\begin{array}{ll} H_{p}/c_{s}, & H_{p}<c_{s}T_{mp}\\ T_{mp}, & c_{s}T_{mp}\leq H_{p}\leq c_{s}T_{mp}+H_{mp}\\ T_{mp}+\left(H_{p}-c_{s}T_{mp}-H_{mp}\right)/c_{m}, & H_{p}>c_{s}T_{mp}+H_{mp} \end{array}$$

(23) Cd(Rep)=24Rep(1+0.179Rep0.5+0.013Rep)

(24) Rep≤103,Rep=2rpρm|V→−V→p|μ

(25) Nup=2+0.459Rep0.55Pr0.33;Nup=(2rpαTp)/km;Pr=μcm/km

where *C_d_* is the drag coefficient, $H_{p}=\int_{T_{0}}^{T_{p}}c_{p}(T)dT$ is the enthalpy of particles, *r_p_* is the particle radius, ${\vec{V}}_{p}$ is the particle velocity, *T_pm_* is the liquidus temperature of the SiC particles, and *H_pm_* is the latent heat of fusion of SiC particles. The heat flux to an individual particle includes heat exchange with the liquid metal matrix.

### 3.6. Boundary Conditions

#### 3.6.1. Energy Boundary Conditions

The energy boundary condition on the top surface of the substrate can be described by the heat input from the arc heat flux and the heat loss from convection and radiation, as shown in Equation (25).

(26) $$k\frac{\partial T}{\partial \vec{n}}={\dot{q}}_{arc}-{\dot{q}}_{conv}-{\dot{q}}_{rad}$$

where $q_{arc}$, $q_{conv}$, $q_{rad}$ are the arc heat input and convective and radiative heat loss, respectively, and $\vec{n}$ is the surface normal.

The molten pool created by the preceding variable polarity TIG welding arc is a prerequisite to ensure that a complete metallurgical bonding layer at the droplets/molten pool interface can be formed. The establishment of a reasonable heat flux field model of the variable polarity TIG welding arc is the key to obtaining accurate numerical calculation results. Based on an in-depth analysis of the TIG-assisted DDM, a dual ellipsoidal heat source model was used as the heat source model of TIG welding molten pool. The numerical simulation process considers the effect of the variable polarity pulse welding current on the flow pattern of the molten pool.

The arc heat input *q_arc_* is given as

(27) $$q_{arc}=\eta UI$$

where *η* is the thermal efficiency of the arc, *U* is the arc voltage, *I* is the arc current.

The heat flux density *q_f_* on the front half axis of the double ellipsoid at (*x*, *y*, *z*) is

(28) $$q_{f}=\frac{6\sqrt{3}q_{arc}f_{f}}{\pi \sqrt{a_{f}bc}}\exp\left(-3[\frac{{\left(x-v_{0}t\right)}^{2}}{a_{f}^{2}}+\frac{y^{2}}{b^{2}}+\frac{z^{2}}{c^{2}}]\right)$$

The heat flux density *q_r_* on the second half axis of the double ellipsoid at (*x*, *y*, *z*) can be calculated as

(29) $$q_{r}=\frac{6\sqrt{3}q_{arc}f_{r}}{\pi \sqrt{a_{r}bc}}\exp\left(-3[\frac{{\left(x-v_{0}t\right)}^{2}}{a_{r}^{2}}+\frac{y^{2}}{b^{2}}+\frac{z^{2}}{c^{2}}]\right)$$

where *a_f_*, *a_r_*, *b* and *c* are the distribution parameters of the arc heat source; *f_f_* and *f_r_* are the heat distribution coefficients of the two parts of the heat source, and *v*_0_ is the arc moving speed, *t* is time.

For single-track deposition SiC-particle reinforced AMCs (the welding torch is at an angle of 65° with the substrate surface), the distribution parameters of the dual ellipsoidal heat source model are DCEN stage: *a_f_* = 4 mm, *a_r_* = 6 mm, *b* = 4 mm, *c* = 1.2 mm, DCEP stage: *a_f_* = 3 mm, *a_r_* = 4 mm, *b* = 3 mm, *c* = 0.9 mm. The model distribution parameters under other process conditions are also determined according to the above method.

For other surfaces:

(30) $$k\frac{\partial T}{\partial n}=h_{A}(T-T_{0})-\sigma \varepsilon_{r}(T^{4}-T_{0}^{4})$$

where *h_A_* is convection coefficient, *σ* and *ε_r_* are Stefan–Boltzmann constant and radiation emissivity, respectively.

#### 3.6.2. Momentum Boundary Conditions

For Marangoni convection, surface tension gradient on the free surface of the molten pool is equal to the shear stress:

(31) $$\begin{array}{c} \mu \frac{\partial u}{\partial z}=-\frac{\partial \gamma }{\partial T}\frac{\partial T}{\partial x}\\ \mu \frac{\partial v}{\partial z}=-\frac{\partial \gamma }{\partial T}\frac{\partial T}{\partial y} \end{array}$$

The normal pressure boundary conditions at the molten pool surface can be expressed as

(32) $$-p+2\mu \frac{\partial {\vec{V}}_{n}}{\partial \vec{n}}=-P_{arc}-\frac{\gamma }{R_{c}}$$

where ${\vec{V}}_{n}$ is the normal velocity, and *R_c_* is radius of the surface curvature.

### 3.7. Numerical Method

Finite volume method was adopted to discrete the related governing equations, the structural finite difference grid was adopted to define the computational domain, and the grid size is 200 μm. Time step is set to be 5 × 10^−6^ s. The explicit method was applied to update the flow fields. The pressure equation (Poisson equation) is solved by Successive Over Relaxation (SOR) method to satisfy the continuity equation, and then, the energy equation was solved by the implicit method. Finally, the VOF equation was used to update the configuration of the free surface. These steps will be repeated at every time step until the specified simulation time is reached.

### 3.8. Properties of As-Used Materials and TIG-Assisted DDM Processing Parameters

The metal matrix feedstock material was a commercial ZL101 aluminum alloy; its chemical composition (mass fraction, %) is Si 6.6%, Mg 0.28%, Fe < 0.16%, Mn < 0.10%, Zn < 0.10%, and Al balanced. The liquidus and solidus temperature of SiC/ZL101 composites were tested by differential scanning calorimetric (DSC) method. The thermophysical properties of the as-used materials in TIG-assisted DDM are listed in Table 1.

The material of substrate was 2024 aluminum alloy (Al-Cu-Mg) in initial T6 condition with a thickness of 6 mm. The chemical composition of the substrate is 4.9Cu-1.8Mg-0.9Mn-0.5Si-0.5Fe-bal.Al (wt%). The material was cut into several pieces with 200 × 200 × 6 mm^3^ dimensions. The initial temperature of the substrate was controlled at 200 °C. A Fronius Magic Wave 3000 welding power source (Fronius International GmbH, Pettenbach, Austria) was adopted. The VP-TIG welding process used was variable-balance square-wave AC mode. Before deposition, oil and other impurities that sit on the substrate surface were removed using acetone. Pure argon (99%) was used as a shielding gas with flow rates of 15~18 L/min for VP-TIG, the working distance is about 6 mm. Initial conditions and the process parameters of TIG-assisted DDM are shown in Table 2. Solidification contact angle is assumed to be constant.

## 4. Experiment Details

### 4.1. TIG-Assisted DDM PROCESS of SiC-Particle Reinforced AMCs

In this study, the experimental material was 4 vol pct SiC reinforced ZL101 (Al-7Si-0.3Mg) composite, a customized TIG-assisted DDM machine was used. The particle size range is between 30 and 72 μm, with an average particle size of 45 μm, obtained using a Malvern Mastersizer 3000E (Malvern, UK). Before TIG-assisted DDM experiment, as-cast SiC/ZL101 composites were machined into feedstocks with mass of 1 kg. The Al-based MMCs was melted in an induction-heated crucible at 720~730 °C and then incubated for 15 min. The AMCs melt was ejected in the form of droplets from the graphite nozzle via piezoelectric ceramic rod. The falling droplets of SiC/ZL101 composites will continuously impact on the molten pool surface, and thus, droplets are arrested, and their final solid shape determined. A CMOS Near Infrared (NIR) camera (XIRIS XVC-1000e) was used to record high-resolution images of the morphology evolution of molten pool surface during droplet impact and the bright welding arc with an exposure time of 80 μs, a frame rate of 30 fps, and a spectral range up to 1500 nm. To reduce the risk of forming adhesions between the impacting AMCs droplets and the tip of the electrode, the location of the droplet impact and the center of molten pool in the horizontal direction was kept at a distance of 2 mm.

### 4.2. Microstructural Characterization

Metallographic examinations were conducted, samples for metallographic observation were cut, ground, and polished according to standard procedures. A solution consisting of HF (2 mL), HCl (3 mL), HNO_3_ (5 mL), and distilled water (180 mL) was taken as an etching agent, with an etching time of 15 s. Microstructural analysis of the dispersions of SiC reinforcing-particles in the ZL101 matrix was performed using a light optical microscope (LOM) (Olympus-BX60M, Tokyo, Japan).

## 5. Results and Discussion

### 5.1. Experiment Verification

**Figure 2:**
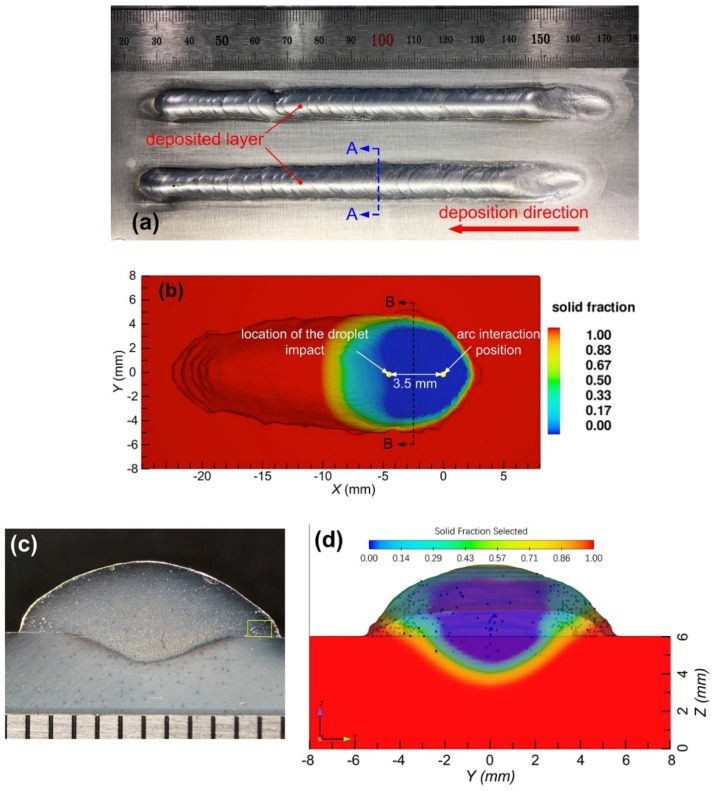
Top surface morphology and cross-section of a single-track deposited layer: (**a**,**c**) experimental results, (**b**,**d**) simulated results.

The surface morphology and cross-section of the as-deposited sample are employed to validate the simulation results. Figure 2a shows the surface morphology of two single-track deposited layers with SiC reinforcing particles, they are formed under the same process conditions as listed in Table 2. The deposition direction is from right to left because the droplet impact position is fixed; the substrate tends to move from right to left.

**Figure 3:**
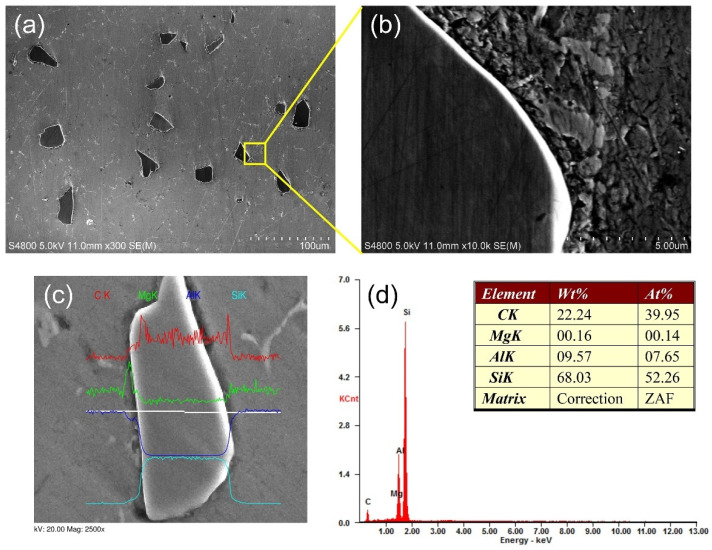
SEM microstructures of ZL101 Al matrix composites in the specified region (Figure 2c): (**a**) AMC-SiC; (**b**) interface between SiC particles and the Al alloy matrix; (**c**) EDS line scanning of the SiC particle and (**d**) EDS spectrums corresponding to the locations in (**c**).

The distributions of the reinforcement particles in the matrix were characterized by using scanning electron microscopy (SEM) equipped with energy dispersive spectrometry (EDS). SiC particles with irregular shapes is no-uniformly distributed in the Al alloy matrix (Figure 3a). Meanwhile, it was observed that there are no agglomerations of SiC particles and free regions in the Al matrix. The interface between the SiC particles and the Al matrix is relatively clear. According to the EDS line scanning results in Figure 3c, light signs of interface reaction were detected.

Table 3 gives the detailed feature sizes (i.e., width, height and the depth of penetration) of the calculated and experimental results from metallographic samples. From Figure 2b, it is clear that the simulated feature sizes of the single-track deposition geometry are in good agreement with experimental results. The relative errors are within 9.5%. A qualitative comparison between the experimental and simulation results for the final distribution state of SiC reinforcing particles on the cross section of the deposited layer is also presented in this figure. Clearly, both the quantitative and qualitative comparisons show that the established numerical model exhibits high accuracy and can be used to reveal the complex physics behind the droplet impact effects on molten pool dynamics and particulate migration behaviors.

### 5.2. Impact, Spreading, and Solidification Behaviors in the TIG-Assisted DDM Process

**Figure 4:**
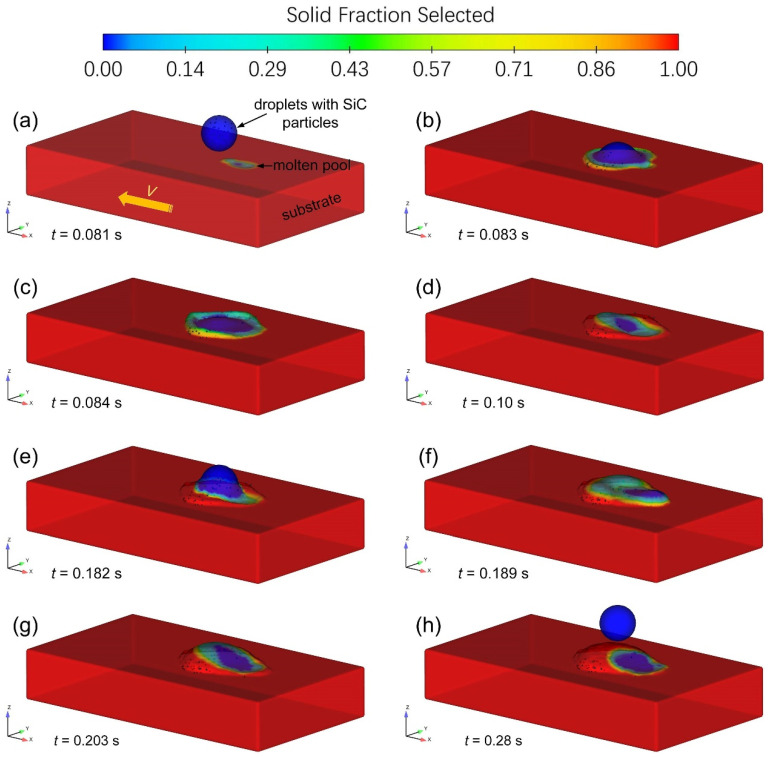
Solid fraction distribution and surface deformation of a single-track deposited layer in the early stage of TIG-assisted DDM.

Figure 4 shows the solid fraction distribution and the surface deformation of a single-track deposited layer in the early stage of TIG-assisted DDM, which are associated with the impact, spreading, and solidification process.

As shown in Figure 4a, a shallow molten pool on the top surface of the moving substrate was firstly created by the fixed preceding TIG welding arc. The surface of molten pool was deformed under the action of arc forces and surface tension force, an obvious depression zone at the front of the molten pool can be seen, the melted metal will flow backward to form a swelling at the rear region of molten pool. As time increased, the successive molten aluminum alloy droplets with SiC reinforcing particles were released from specified heights, *H*, with a vertical velocity of 1.2 m/s. When a molten aluminum alloy droplet impacts on the rear region of molten pool, it coalesces firstly with the receiving liquid metal with particle reinforcement, as shown in Figure 4b. Then, most of the melt will rapidly spread on high-temperature solid surface around the molten pool. As shown in Figure 4c, a liquid crown will be visible above the surface. As the deposition process continues, the solidified microstructure in the deposition area is continuously moved from right to left under the action of the horizontally moving substrate (Figure 4d). The introduction of droplet energy leads to an increase in local heat accumulation, which in turn increases the size of the molten pool, the corresponding results are shown in Figure 4e–h.

### 5.3. Migration Behaviors of the SiC Particles in the Molten Pool

**Figure 5:**
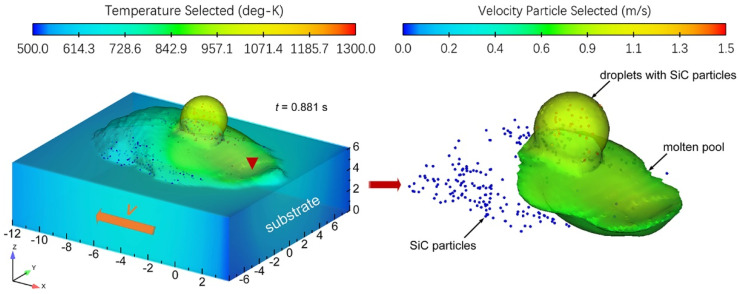
Distribution position of SiC particle-reinforced phase in the deposited layer.

During the deposition process, there are complex two-way coupling drag forces between the SiC particle-reinforced phase and the molten aluminum matrix. At the same time, the annexation and repulsion of the interface between the reinforced phase and the solidified phase of SiC particles directly affect the distribution characteristics of the enhanced phase in the solidified tissue. Figure 5 shows the distribution position of the SiC particle-reinforced phase in the deposited layer.

It can be seen from Figure 5 that in the solidified microstructure of the deposited layer, the SiC particle-reinforced phase is mainly concentrated on both sides of the sedimentary layer due to the flow of the molten pool and the drag force of the melt. It is consistent with the experimental results. It is proved that the molten pool thermal flow field calculation model established in this paper can provide a theoretical basis for the quantitative study of heat and mass transport and solidification behavior in the TIG-assisted DDM of SiC particle-reinforced AMCs components.

**Figure 6:**
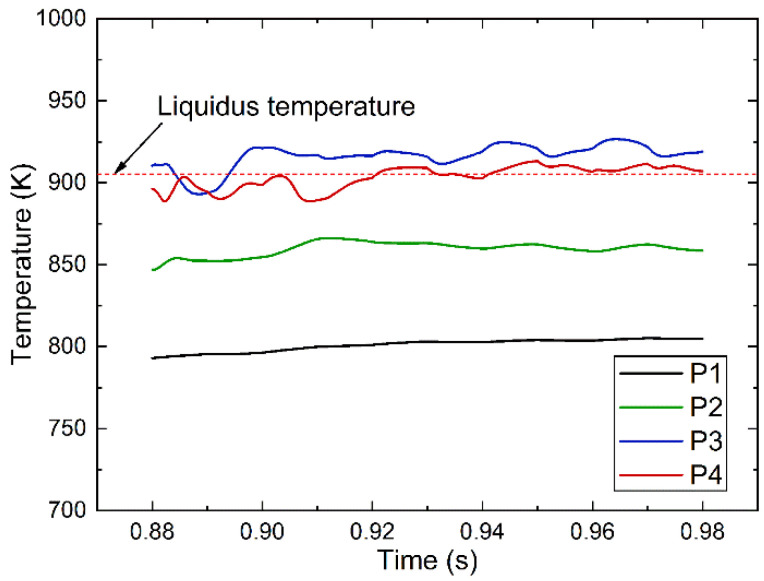
Temperature evolution at specified points (P1, P2, P3 and P4) during one deposition period.

Figure 6 demonstrates the temperature evolution of four fixed points during one deposition period. Here, the vertical distances from P1, P2, P3, P4 to the top surface of substrate are all kept at 2 mm, the coordinates of the four points are P1(−6, 0, 2), P2(−4.5, 0, 2), P3(−3, 0, 2) and P4(0, 0, 2). Point P4 is located below the arc action position.

It was clear that the magnitude of temperature fluctuation at the specified points was found to be closely related to the molten pool behavior induced by the droplet impact. During coalescence, after droplet impact the force is more towards the crater center than the crater sidewalls, and thus, the thermal energy of the impacting droplet is absorbed and dissipated by the molten. On the other hand, the temperature at the four points tend to decrease when the incoming droplet start to contact the molten pool. Therefore, a decrease in the temperature at the points can be seen. However, the magnitude of temperature fluctuation at point P3 are the more obvious, which can be attributed to the flow oscillation and the thermal energy variation induced by the impacting droplet and the variable polarity gas tungsten arc welding power.

## 6. Conclusions

In this study, a 3D numerical model based on the finite volume method was established to clarify the mechanism of the particulate migration and the molten pool behaviors induced by successive droplet impact and variable-polarity TIG welding arc. The resultant surface morphology of the AMCs deposited layer and the distribution state of SiC particle reinforcement were experimentally acquired, which are in a good agreement with the results predicted by simulation. The main conclusions can be summarized as follows:

(1) The feasibility to fabricate ceramic-reinforced aluminum matrix composite components by TIG-assisted DDM has been proven.
(2) The cross-sectional morphology of a single-track deposited layer and the distribution characteristics of the SiC particle-reinforced phase reasonably agree with the experimental results, and the relative errors are within 9.5%, which indicated that the model can reveal the formation mechanism of TIG-assisted DDM.
(3) The non-uniformly distribution of SiC reinforcing particles on the cross-section of deposited layer could be found, which can be attributed to the viscous dissipation effect and the time-delayed velocity difference during the droplet spreading and recoiling stage.

## Figures and Tables

**Table 1:** The relevant thermophysical properties of SiC particle and ZL101 aluminum alloy.

Thermal Properties	Value
Solid density of ZL101	*ρ_s_* = 2500 (kg·m^−3^)
Liquid density of ZL101	*ρ_l_* = 2420 (kg·m^−3^)
Solidus temperature	*T_s_* = 829 (K)
Liquidus temperature	*T_l_* = 905 (K)
Latent heat of fusion	*L* = 4.29 × 10^5^ (J·kg^−1^)
Latent heat of evaporation	*L_ev_* = 2.8 × 10^7^ (J·kg^−1^)
Thermal conductivity of solid phase	*k_s_ =* 167 (W·m^−1^·K^−1^)
Thermal conductivity of liquid phase	*k_l_ =* 79.5 (W·m^−1^·K^−1^)
Specific heat capacity of solid phase	*C_s_ =* 1194 (J·kg^−1^·K^−1^)
Specific heat capacity of liquid phase	*C_l_ =* 1265 (J·kg^−1^·K^−1^)
Dynamic viscosity	*ν =* 0.008 (Pa·s)
Surface tension	*γ*_0_*=* 0.871 (N·m^−1^)
Temperature coefficient of surface tension	*k*_sur_ = −3.5 × 10^−4^ (N·m^−1^·K^−1^)
Thermal expansion coefficient	*ρ_T_* = 1.5 × 10^−4^ (K^−1^)
Particle density	*ρ_p_* = 3100 (kg·m^−3^)
Particle diameter	*r_p_* = 40 (m^−6^)
Particle fraction	*f =* 0.04
Thermal conductivity of particle	*k_p_ =* 120 (W·m^−1^·K^−1^)
Specific heat capacity of particle	*C_p_ =* 750 (J·kg^−1^·K^−1^)
Liquidus temperature of particle	*T_pm_* = 1955 (K)
Latent heat of fusion of particle	*H_pm_* = 3.7 × 10^5^ (J·kg^−1^)
Ambient temperature	*T*_0_ = 298 (K)
Magnetic permeability	*μ*_0_ = 1.26 × 10^−6^ (H·m^−1^)
Convection heat transfer coefficient	*h_c_* = 40 (W·m^−2^·K^−1^)
Radiation emissivity	*ε* = 0.4
Stefan-Boltzmann constant	σ = 5.67 × 10^8^ (W·m^−2^·K^−4^)

**Table 2:** Initial conditions and the process parameters of TIG-assisted DDM.

Process Parameters	Value
EP ratio	50 (%)
Current cycle	*t*_0_ = 20 (ms)
Arc current in EN phase	*I*_en_ = 100 (A)
Arc current in EP stage	*I*_ep_ = 200 (A)
Arc voltage at EN stage	*U*_en_ = 14.3 (V)
Arc voltage at EP stage	*U*_ep_ = 12.1 (V)
Arc heat absorption	*η*_en_ = 0.7
	*η*_ep_ = 0.4
Substrate moving speed	*v* = 7 (mm s^−1^)
Torch angle	*θ* = 65 (°)
Droplet temperature	*T_d_* = 1020 (K)
Droplet diameter	*d*_0_ = 3 (mm)
Droplet frequency	*f* = 10 (Hz)
Impact velocity of droplets	*v*_0_ = 1.2 (mm)

**Table 3:** Comparison of calculated and experimental feature sizes of a single-track deposited layer.

	Simulation	Experiment	Error
Deposition width	10.6 mm	11.2 ± 0.3 mm	5.4%
Deposition height	3.1 mm	3.3 ± 0.2 mm	6.1%
Depth of penetration	1.2 mm	1.1 ± 0.1 mm	9.1%

## Data Availability

The data presented in this study are available on request from the Corresponding author.
